# Comparative Evaluation of *qnrA*, *qnrB*, and *qnrS* Genes in *Enterobacteriaceae* Ciprofloxacin-Resistant Cases, in Swine Units and a Hospital from Western Romania

**DOI:** 10.3390/antibiotics9100698

**Published:** 2020-10-14

**Authors:** Alexandru O. Doma, Roxana Popescu, Mihai Mitulețu, Delia Muntean, János Dégi, Marius V. Boldea, Isidora Radulov, Eugenia Dumitrescu, Florin Muselin, Nikola Puvača, Romeo T. Cristina

**Affiliations:** 1 Faculty of Veterinary Medicine, Banat’s University of Agricultural Sciences and Veterinary Medicine Timișoara, Calea Aradului 119, 300645 Timișoara, Romania; dao_west@yahoo.com (A.O.D.); janosdegi@usab-tm.ro (J.D.); marius_boldea@usab-tm.ro (M.V.B.); isidoraradulov@yahoo.com (I.R.); eugeniadumitrescu@usab-tm.ro (E.D.); florinmuselin@usab-tm.ro (F.M.); 2 Department of Cellular and Molecular Biology, University of Medicine and Pharmacy Timișoara, Piaţa Eftimie Murgu, 2, 300041 Timişoara, Romania; popescu.roxana@umft.ro (R.P.); mihai.mituletu@umft.ro (M.M.); muntean.delia@umft.ro (D.M.); 3 Department of Engineering Management in Biotechnology, Faculty of Economics and Engineering Management in Novi Sad, University Business Academy in Novi Sad, Cvećarska 2, 21000 Novi Sad, Serbia

**Keywords:** *Enterobacteiaceae*, ciprofloxacin-resistant, *qnrA*, *qnrB*, *qnrS*, genes

## Abstract

Excessive use of antimicrobials and inadequate infection control practices has turned antimicrobial resistance (AMR) into a global, public health peril. We studied the expression of *qnrA*, *qnrB*, and *qnrS* plasmid in ciprofloxacin (CIP)-resistant strains of *Escherichia coli* in swine and humans from Romania, using the Polymerase Chain Reaction (PCR) technique. Antibiotic Susceptibility Testing (AST) for human subjects (H) on 147 samples and 53 swine (S) was ascertained as well as the isolation of bacterial DNA (*E. coli*) as follows: bacteriolysis, DNA-binding, rinsing, elution, amplification, and nucleic acids’ migration and U.V. visualization stages. From 24 samples of *E. coli* resistant to CIP collected from H subjects and 15 from S, for PCR analysis, 15 H and 12 S were used, with DNA purity of 1.8. The statistically analyzed results using the *Crosstabs* function (IBM SPSS Statistics-Ver. 2.1.), revealed the *qnrS* (417 bp) gene in 13 human subjects (52.0%), as well as in all swine samples studied. The *qnrB* (526 bp) gene was exposed in 9 of the human patients (36.0%) and in all swine isolates, and the *qnrA* (516 bp) gene was observed only in 3 of the isolates obtained from human subjects (12.0%) and was not discovered in pigs (*p* > 0.05). The presence of plasmids *qnrA*, *qnrB*, and *qnrS* in the human samples and of *qnrB* and *qnrS* in swine, facilitates the survival of pathogens despite the CIP action. The long-term use of CIP could cause a boost in the prevalence of *qnr* resistance genes, and resistance in the pigs destined for slaughter, a perturbing fact for public health and the human consumer.

## 1. Introduction

Antimicrobial resistance (AMR) is the capacity of microorganisms to adjust to antimicrobials, particularly antibiotics. Excessive and improper uses of antimicrobial drugs and inadequate infection control practices have turned AMR into a severe global public health peril [1,2].

According to the European Commission (EC), the influence of the imprudent use of anti-infectives is substantial. Thus, more than 70% of bacteria, accountable for intra-hospital infectivity, were found to be resistant to at least one antibiotic structure. AMR is also responsible for more than 25,000 human deaths/year in the EU, and 700,000 worldwide, and might lead to more deaths than cancer by 2050 [3,4].

In this respect, databases and surveillance systems, from both the human health and veterinary sector, are becoming increasingly ample in data, since resistance was reported for nearly all antibiotic structures. In Romania, the main indicator for antimicrobial consumption in the veterinary sector is the Population Correction Unit (PCU), who revealed that the consumed amount of antibiotics was 100.5 mg × PCU^−1^, an almost identical value with the EU average (100.6 mg × PCU^−1^) in 2015 [2,5]. Between 2010 and 2030, global antimicrobial consumption in the livestock sector is expected to increase by approximately 70%, however, only a quarter of countries have implemented a national policy to combat AMR [5].

Though the antimicrobials have greatly modernized current medical practices, today, this advantage is particularly at risk due to intense or improper use of antimicrobials. The irresponsible use of antibiotics has amplified the occurrence and spread of multidrug-resistant bacteria, making optimization of veterinary antimicrobial treatment a priority [6].

Along with antibiotics used in human medicine, their use for treatment or prophylaxis practices used in animal breeding have led to selective pressure, favoring the emergence and rapid spread of resistant bacterial strains [7,8,9]. In this aim, animals can serve as mediators, reservoirs, and disseminators of resistant bacterial strains and/or resistance genes. Multiple studies have reported that excessive or inadequate use of antimicrobial substances in animals destined for production in the food industry can have a negative impact on the health of hired farm workers, of employees from meat processing units, as well as the on the final consumer [7,8,9].

The link between antibiotic and antimicrobial resistance has already been statistically demonstrated for *Escherichia coli* resistant to fluoroquinolones in humans [10,11] or animals [12,13,14], and also, *E. coli* resistant to cephalosporins from third and fourth generation in humans, *E. coli* resistant to tetracyclines and polymyxins in animals, *Klebsiella pneumoniae* resistant to carbapenems and polymyxins in humans, and *Campylobacter* spp. strains that are resistant to macrolides from animals associated with cross-resistance in animals and humans. Multiple resistances have also been reported in *Salmonella typhimurium* strains to antibiotics such as ampicillin, chloramphenicol, streptomycin, sulfonamides, and tetracycline [3,4,9,12,15].

The quick identification and antimicrobial susceptibility testing have considerable effects on the clinical outcome of severe infections in humans and animals. The frequent emergence of resistance to quinolones occurring in common infections with *Campylobacter* spp. and *E. coli* in humans, as a result of their massive use in animal feed, as well as the transmission of human-resistant bacteria through meat and animal products, causes great awareness [8,13,14].

Fluoroquinolones impede DNA gyrase and topoisomerase IV enzymes, both with crucial roles in bacterial DNA replication, and resistance to quinolones is regularly associated to amino-acid substitutions of *gyrA* and *gyrB* gyrases, DNA topoisomerase IV subunits, the quinolone-resistance-determining regions, followed by target modification [16,17].

Quinolones group have been used for prophylaxis against Gram-negative infections both in humans and animals, but the impact on the resistance mechanisms of this important group nonetheless require additional exploration [18,19].

The *qnr* genes provide low resistance level to quinolones in *Enterobacteriaceae*, but the multi-resistance dimension is of great importance, and studies about the resistance of *E. coli* to ciprofloxacin (CIP) and the specific *qnrA*, *qnrB*, and *qnrS* genes’ detection and expression have been published in the last years [20,21,22].

In these cases, the use of Polymerase Chain Reaction (PCR) offers a simple, rapid, and accurate detection of the antibiotic resistance profiles, becoming a regularly used method of antibio-resistance diagnosis and surveillance in the epidemiological and ecological studies [23,24,25].

Since we were concerned with the quinolone group’s resistance, emergent in Western Romania, to humans and animals, the present study tried to identify CIP-resistant cases and monitored the *qnrA*, *qnrB*, and *qnrS* plasmids in *E. coli*, using the molecular technique. The aim was to analyze the extent to which these ciprofloxacin resistance genes are present, and to examine their clone relatedness in pigs and human samples from our region.

## 2. Results

### 2.1. Antibiotic Susceptibility Testing (AST)

The results obtained from AST and the evolution of the resistance tendency showed the considerable presence of the multi-resistant strains in swine isolates where, from 53 samples analyzed, 15 isolates were presenting resistance to CIP and also multi-resistance to other antibiotics, including other quinolone representatives like, enrofloxacin, in the majority of cases, and norfloxacin (Table 1).

The AST results obtained from 147 human samples also presented high CIP resistance levels, but proportionally lower compared to swine, with 38 isolates presenting resistance, and among these, 17 were found with resistance for more than two antibiotics (Table 2).

Crosstabs function and statistics for human and swine samples are presented in Table 3 and Table 4.

From a statistical point of view and according to the obtained results, percentage 0.0% should be less than 20% and 0.000 less than *p* > 0.05, so the null hypothesis is rejected, meaning there are significant differences between antibiotics with an error of *p* > 0.05.

### 2.2. PCR Techniques—Isolation of Bacterial DNA (E. coli) in Humans and Swine

For PCR analysis, only samples with DNA purity of approximately 1.8 were processed, with the values recorded below this level signifying the samples’ contamination. Thus, from the *E. coli* cultures collected for PCR analysis from humans (H), we used 15 DNA samples from a total of 24 taken from the culture media, and of 15 swine (S) samples studied, only 12 had quantitatively and qualitatively appropriate genetic material.

Following the isolation, we carried on with the migration of the DNA in agarose gel for the additional verification of the genetic material’s integrity.

The extent to which CIP resistance genes (*qnrA*, *qnrB*, and *qnrS*) were present in the bacterial genome isolated from pigs and human subjects is presented in Table 5.

The presence of the *qnrS* gene (417 base pairs—bp) was identified in 13 of the human subjects and in all pigs registered in our study.

PCR amplification for the *qnrB* gene (526 bp) showed its presence in 9 of the human patients and in all cases of isolates obtained from pigs.

The *qnrA* gene (516 bp) was observed only in 3 of the isolates obtained from human subjects, but it was absent in pig isolates.

Accordingly, in cultures of *E. coli* isolated from human samples, *qnrS* was detected in 52%, *qnrB* in 36%, and *qnrA* in 12% of cases, respectively. Similarly, in swine samples, *qnrS* and *qnrB* were reported in 100% of swine samples but no *qnrA* genes were reported. The obtained results point out an increased prevalence of *qnr* resistance genes in CIP-resistant *E. coli.* A differentiation between the two situations studied is the presence of *qnrA* genes only in humans. This leads to the assumption of direct or indirect contact of these subjects with low concentrations of CIP, which may increase resistance through the presence of plasmid *qnrA*, a mechanism that facilitates the survival of pathogenic *E. coli* germs.

## 3. Discussion

The introduction of ciprofloxacin in the therapeutic protocols of the 80′s represented a real progress for the medicine of those times. After only a decade of use, unfortunately, the first cases of resistance appeared, with much lower incidence compared to current times [26,27,28].

The expansion of this phenomenon over time may coincide with the massive detection of *qnr* genes. This has been a suspicion of various researchers due to the close links between *qnr* genes and diverse quinolones resistance. It has been demonstrated through “in vitro” procedures on *qnrA* plasmid, which facilitated the development of this phenomenon in *Enterobacteriaceae*, at the chromosomal level [29,30,31,32].

The present study confirmed an increased prevalence of *qnrA*, *qnrB*, and *qnrS* resistance genes in quinolones in both human and swine subjects, and the presence of *qnrA* genes at a 12% rate in humans only stands as a differentiation between the two situations analyzed. This leads to the hypothesis regarding direct or indirect contact of these subjects with low concentrations of ciprofloxacin, which may increase resistance through the presence of plasmid *qnrA*—a mechanism that facilitates the survival of pathogenic *E. coli* [31,32].

After testing the pigs, however, some significant differences of the resistance phenomenon can be observed and confirmed through statistical interpretation of the results using the Crosstabs function. The presence of these differences can be based on the following assumptions:

- Dissimilar evolution of bacterial diseases on farms,
- Diverse treatment protocols between units,
- Organization of antimicrobial products through treatment rotation.

A simple comparison between the values obtained by us in the hospital in Timișoara and the clinical units in other areas of the world, show the increased incidence of quinolone-resistant *Enterobacteriaceae*: at a 32% rate in UK—Liverpool [12], and at an overwhelming rate of 78% genes encoded by *qnrA* in the Netherlands [10].

In the USA, the presence of the *qnrA* and *qnrB* genes only, was also reported [15]. In Taiwan and Korea, the presence of these genes was around 17% (*qnrA* 0.6%, *qnrB* 10%, and *qnrS* 6.5%) [11,32].

After analyzing this situation in other hospitals, from the first discovery of a quinolone resistance gene in 1998, until now, we can state that the evolution of this phenomenon differs greatly, depending on the area and the therapeutic protocols. From a genotypic and structural point of view, it is known that the composition of the two genes includes 218 amino acids with a variety lower than 10% between *qnrA* and *qnrS*. Similarities between *qnrB* and *qnrA* are of only 40%, with the first being composed of 214 amino acids [25,32].

The situation of the Romanian farms studied does not differ much from those in China, in terms of gene type presence. In both cases, the *qnrA* gene is missing, and as a differentiation, the incidence of *qnrB* plasmids is lower in Chinese farms. In the case of pigs from Chinese households, the presence of resistance genes is around 6%. Furthermore, in swine farms from Taiwan, the presence of the *qnrS* gene was reported to be around 3.33% [31].

It is general knowledge today that the long-term use of quinolones was followed by an increase in prevalence of *qnr* resistance genes, and cases of resistance have also been reported in pigs. Also, in our study, we ascertained an associated antibio-resistance of CIP with enrofloxacin and norfloxacin, which confirmed the multi-resistance high tendency for the quinolones group. Thus, some alarm signals were raised about the zoonotic transmission of this phenomenon through the food chain [3,5,7,8,9,24,25].

Healthcare organizations, as well as recent research, have focused on the global assessment of this phenomenon of resistance. The impact of much more restrictive protocols on the handling and use of antimicrobials has already led to a trend of significant percentage decrease in resistance—between 9% and 30%. In order to avoid the propagation of this phenomenon, the precautionary principle is recommended. The legislative revision of used therapeutic protocols, by establishing new limitations on the handling and use of antimicrobials, has become an absolute priority [33].

## 4. Materials and Methods

### 4.1. Location and Samples Collecting for AST

The study was conducted over one year (from Jan 2019–Jan 2020).

The experiment took place in the Western part of Romania, in Timiș and Arad Counties, areas well developed from the perspective of swine breeding, with an annual population of over one million pigs, in the intensive system only. A big part of this production is destined for meat consumption, as well as meat-derived products. For the purpose of this research, we included large capacity swine exploiting units, where clinical cases were diverse, and the incidence of colibacillary infections was high.

#### 4.1.1. Samples

The examination was performed on biological material, from pure *E. coli* cultures (of maximum 20 mg), collected directly from the fresh intestinal contents of swine.

The samples from humans were provided for the lab processing from a large hospital from Timișoara city, Romania, with the blood samples being gathered in blood collection K3-EDTA vacutainer tubes (13 × 100 mm) (Kima, Bucharest, Romania).

#### 4.1.2. Microbial Testing

Subsequently, bacterial resistance of 147 isolated *E. coli* strains from humans and 53 for swine were tested for susceptibility to twenty-one commonly used antibiotics in human medicine and fifteen frequently utilized antibiotics and associations, through the Kirby–Bauer standardized disk diffusion technique.

Interpretation of antibiotic resistance was performed through measuring the diameter of the growth inhibition zone and the strains were categorized as Non-aligned (Intermediary sensitive), Resistant, or Sensitive to the drug according to manufacturer’s instructions and according with the current interpretation standards, which can be found in the Clinical Laboratory Standards Institute (CLSI), Performance Standards for Antimicrobial Disk Susceptibility Tests.

### 4.2. PCR Techniques—Isolation of Bacterial DNA (E. coli) in Humans and Swine Samples

Samples were taken in Phosphate Buffered Saline (PBS) solution (Sigma-Aldrich, Darmstadt, Germany) to culture plates and cultivation of *E. coli* strains was on McKonkey (Oxoid Ltd., Basingstoke, UK) selective media, then *E. coli* was sampled in tubes for PCR analysis.

Bacterial DNA isolation was performed using the PureLink^®^ Genomic DNA Mini Kit (Invitrogen, Carlsbad, CA, USA) according to the manufacturer’s protocol.

Analysis of the quality and quantification of DNA extracted from bacterial cultures was performed using UV spectroscopy. For appreciating DNA purity, we analyzed the Optical Density (OD) at 260/280 on a ScanDrop nano-volume spectrophotometer (Analitik Jena, Jena, Germany).

For PCR analysis, we took into account only samples with DNA purity of approximately 1.8, with the values recorded below this level indicating the contamination of samples.

DNA amplification was performed in PCR on a thermo-cycler (BiometraTM, Analitik Jena, Jena, Germany), for 35 cycles, using FIRESol^®^ Master Mix (Solis BioDyne, Tartu, Estonia) and specific primers for *qnrS*, *qnrA*, and *qnrB* genes.

Work protocol used 500 µL tubes to obtain a 50 µL reaction volume, by adding 45 µL PCR mix and 5 µL primers and 1:1 DNA sample. Multiplex reagents were performed for *qnr* analysis, and sequence of primers used, genes, and fragment size are shown in Table 6.

### 4.3. Statistical and Data Analysis

Statistical analysis of data obtained in the experiment regarding the use of antibiotics in the swine units was performed using the IBM SSPS Statistics (Version 2.1.) and Crosstabs function, where 0.000 was less than *p* > 0.05, so the null hypothesis is rejected, i.e., there are significant differences between antibiotics with an error of *p* > 0.05.

## 5. Conclusions

After evaluating this case, we can state that the main *qnrA* gene (516 bp) was not found in swine. Moreover, the presence of *qnrA* (12%), *qnrB* (36%), and *qnrS* (52%) genes in human samples and of *qnrB* and *qnrS* in swine can facilitate the survival of pathogens under the action of antimicrobials from the quinolone group, in our case, CIP alone, or CIP-associated multiple-resistance, both in veterinary practice and in human hospitals’ therapeutic protocol.

Thus, the hypothesis of transmitting resistance on the human-animal-human food chain is demonstrated. The long-term use of CIP could lead to an increase in the prevalence of *qnr* resistance genes, and resistance emergence in the healthy pigs destined for slaughter.

## Figures and Tables

**Table 1:** Swine strains found with multiple resistances to CIP, 15 isolates from a total of 53.

No.	CIP-Resistant Sample No.	Antibiotics Where Resistance Was Identified	Total Antibiotics
1.	S.2.	CIP; NOR; FLO; AMX; CEF; SPCM; TC	7
2.	S.6.	CIP; ENR; AMX; CEF; OXA; FLO; SPCM	7
3.	S.7.	CIP; AMX; OXA; CEF; SPCM; TC	6
4.	S.13.	CIP; ENR; NOR; AMX; FLO; CEF; SPCM; TC	8
5.	S.14.	CIP; AMX; FLO; CEF; TC	5
6.	S.16.	CIP; NOR; AMX; FLO; CEF; SPCM; TC	7
7.	S.22.	CIP; ENR	2
8.	S.28.	CIP ENR; AMX; PSTR; NEO; CST; TC	7
9.	S.35.	CIP; ENR AMX; FLO; LCM; NEO; TC	7
10.	S.36.	CIP; ENR; FLO; AMX; LCM; NEO; TC	7
11.	S.38.	CIP; ENR; AMP; AMX; FLO; LCM; NEO; TC	8
12.	S.46.	CIP; ENR; AMP; GEN; NEO; FLO; LCM; TC; POS	9
13.	S.47.	CIP; ENR; AMP; GEN; NEO; FLO; LCM; TC; POS	9
14.	S.49.	CIP; ENR; AMP; AMX; NEO; STR; GEN; FLO; LCM; TC; POS	11
15.	S.50.	CIP; ENR; AMP; AMX; NEO; STR; FLO; LCM; TC; POS	10

Legend: S—Swine sample; Amoxicillin—AMX; Ampicillin—AMP; Cefalothin—CEF; Ciprofloxacin—CIP; Colistin—CST; Enrofloxacin—ENR; Florfenicol—FLO; Gentamicin—GEN; Lincomycin—LCM; Neomycin—NEO; Norfloxacin—NOR; Oxacillin—OXA; Penicillin-streptomycin—PSTR; Potentiated sulfonamides—POS; Spectinomycin—SPCM; Streptomycin—STR; Tetracycline—TC.

**Table 2:** Human strains found with multiple resistances to CIP, 38 isolates from a total of 147.

No.	CIP -Resistant Sample No.	Antibiotics Where Resistance Was Identified	Total Antibiotics	No.	CIP -Resistant Sample No.	Antibiotics Where Resistance Was Identified	Total Antibiotics
1.	H.6.	CIP; LVX; PIP; SAM; GEN	5	20.	H.78.	CIP; PIP; SAM; CAZ; CTX; CFPM; TZP	7
2.	H.13.	CIP; LVX; TPM	3	21.	H.79.	CIP, PIP	2
3.	H.15.	CIP; LVX; PIP	3	22.	H.80.	CIP, LVX; PIP	3
4.	H.16.	CIP; LVX; PIP; TPM	4	23.	H.85.	CIP; PIP; CTX; CXM	4
5.	H.19.	CIP	1	24.	H.88.	CIP; LVX; PIP; CXM; TPM	5
6.	H.20.	CIP, LVX	2	25.	H.90.	CIP; LVX; GEN; PIP	4
7.	H.21.	CIP, LVX; PIP	3	26.	H.94.	CIP; PIP; CAZ; CTX; CXM; TPM	6
8.	H.22.	CIP; PIP; CAP; CTX; CXM; GEN	6	27.	H.97.	CIP; PIP; SAM; TPM	4
9.	H.24.	CIP; PIP; CTX; TPM	4	28.	H.102.	CIP; PIP; CAZ; CXM	4
10.	H.26.	CIP, LVX	2	29.	H.104.	CIP; PIP; CAZ. CTX; CFPM; TPM	6
11.	H.32.	CIP; PIP; SAM; CTX; CXM	5	30.	H.106.	CIP; PIP.	2
12.	H.35	CIP; PIP; CXM; TPM	4	31.	H.110.	CIP; PIP; TPM	3
13.	H.49.	CIP; LVX; PIP; CTX; CXM	5	32.	H.116.	CIP; LVX; PIP; CXM; TPM	5
14.	H.50.	CIP; LV	2	33.	H.119.	CIP; PIP; SAM; TPM	4
15.	H.60.	CIP; PIP; TPM	3	34.	H.130.	CIP; LVX; SAM; CAZ; CTX; CFPM; GEN; TZP	8
16.	H.65.	CIP; LVX; PIP; SAM; CAZ; CTX; CFPM; GEN	8	35.	H.134.	CIP; TPM.	2
17.	H.68.	CIP; LVX	2	36.	H.142.	CIP; CXM.	2
18	H.70.	CIP; LVX; PIP; CTX; CXM	5	37.	H.144.	CIP; TPM	2
19	H.71.	CIP; PIP; CTX; CXM; TPM	5	38.	H.147.	CIP; PIP; SAM; TPM.	4

Legend: Human sample—H; Ampicillin-sulbactam—SAM; Cefepime—CFPM; Ceftazidime—CAZ; Ceftriaxone—CTX; Cefuroxime—CXM; Ciprofloxacin—CIP; Gentamicin—GEN; Levofloxacin—LVX; Piperacillin—PIP; Piperacillin-tazobactam—TZP; Trimethoprim—TPM.

**Table 3:** Antibiotic Susceptibility Testing (AST) results for human subjects (H) and swine (S).

Cross-Tabulation Results											
Humans (H)						Swine (S)					
Antibacterial		Results			Total	Antibacterial		Results			Total
		N	R	S				N	R	S	
Amikacin	count	4	1	142	147	Amoxicillin	count	6	43	4	53
	%	2.7	0.7	96.6	100.0		%	11.32	81.13	7.55	100.0
Ampicillin-sulbctam	count	20	14	113	147	Ampicillin	count	44	9	0	53
	%	13.6	9.5	76.9	100.0		%	83.02	16.98	0.0	100.0
Aztreonam	count	145	0	2	147	Sulfadoxin	count	49	0	4	53
	%	98.6	0.0	1.4	100.0		%	92.45	0.0	7.55	100.0
Cefepime	count	5	5	137	147	Cefalotin	count	36	17	0	53
	%	3.4	3.4	93.2	100.0		%	67.92	32.08	0.0	100.0
Cefoperazona-sulbactam	count	146	0	1	147	Ceftiofur	count	41	0	12	53
	%	99.3	0.0	0.7	100.0		%	77.35	0.0	22.65	100.0
Ceftazidime	count	6	9	132	147	Ciprofloxacin	count	22	15	16	53
	%	4.1	6.1	89.8	100.0		%	41.51	28.30	30.19	100.0
Ceftriaxone	count	11	15	121	147	Cefquinome	count	46	0	7	53
	%	7.5	10.2	82.3	100.0		%	86.79	0.0	13.21	100.0
Cefuroxime	count	11	21	115	147	Colistin	count	22	14	17	53
	%	7.5	14.3	78.2	100.0		%	41.51	26.42	32.07	100.0
Ciprofloxacin	count	4	39	104	147	Doxycycline	count	37	10	6	53
	%	2.7	26.5	70.7	100.0		%	68.81	18.87	11.32	100.0
Colistin sulphate	count	36	0	111	147	Enrofloxacin	count	16	13	24	53
	%	24.5	0.0	75.5	100.0		%	30.19	24.53	45.28	100.0
Gentamicin	count	10	6	131	147	Erythromycin	count	39	12	2	53
	%	6.8	4.1	89.1	100.0		%	73.58	22.65	3.77	100.0
Imipenem	count	0	0	147	147	Florfenicol	count	5	27	21	53
	%	0.0	0.0	100.0	100.0		%	9.43	50.95	39.62	100.0
Levofloxacin	count	10	19	118	147	Gentamycin	count	4	8	41	53
	%	6.8	12.9	80.3	100.0		%	7.55	15.09	77.36	100.0
Meropeneme	count	3	0	144	147	Lincomycin	count	27	15	11	53
	%	2.0	0.0	98.0	100.0		%	50.95	28.30	20.75	100.0
Minocycline	count	145	0	2	147	Neomycin	count	19	30	4	53
	%	98.6	0.0	1.4	100.0		%	35.85	56.60	7.55	100.0
Ofloxacime	count	145	0	2	147	Norfloxacin	count	36	6	11	53
	%	98.6	0.0	1.4	100.0		%	67.92	11.32	20.75	100.0
Piperacillin-tazobactam	count	16	4	127	147	Oxacillin	count	36	4	13	53
	%	10.9	2.7	86.4	100.0		%	67.92	7.55	24.53	100.0
Piperacillin	count	9	71	67	147	Penicillin-Streptomycin	count	49	3	1	53
	%	6.1	48.3	45.6	100.0		%	92.45	5.66	1.89	100.0
Ticarcillin	count	145	0	2	147	Spectinomycin	count	36	13	4	53
	%	98.6	0.0	1.4	100.0		%	67.92	24.53	7.55	100.0
Tobramycin	count	145	0	2	147	Streptomycin	count	44	7	2	53
	%	98.6	0.0	1.4	100.0		%	83.02	13.21	3.77	100.0
Trimethoprim	count	28	42	77	147	Sulfonamides	count	44	8	1	53
	%	19.0	28.6	52.4	100.0		%	83.02	15.09	1.89	100.0
Total		1044	246	1497	3087	Tetracycline	count	16	31	6	53
							%	30.19	58.49	11.32	100.0
						Tiamulin	count	36	3	14	53
							%	67.92	5.66	26.42	100.0
						Total		710	288	221	1219

Legend: N = Non-aligned (Intermediary sensitive), R = Resistant, S = Susceptible.

**Table 4:** Statistical results of *E. coli* resistance to CIP.

Humans (H)										Swine (S)								
Chi-Square Tests		Value		*df*		Asymp. Sig. (2-sided)				Value		*df*		Asymp. Sig. (2-sided)				
Pearson Chi-Square		2925.127 ^a^		40		0.000				574.795^a^		44		0.000				
Likelihood Ratio		3113.083		40		0.000				588.576		44		0.000				
0 cells (0.0%) have expected count less than 5.0. The minimum expected count is 11.71.										0 cells (0.0%) have expected count less than 5.0. The minimum expected count is 9.61.								
Case Processing Summary	Valid				Missing			Total		Valid			Missing			Total		
	3087		100.0%		0		0.0%	3087	100.0%	1219	100.0%		0		0.0%	1219	100.0%	

Legend: *df*—degree of freedom; Asymp. Sig.—Asymptotic Significance; ^a^—with statistical significance (*p* > 0.05).

**Table 5:** Presentation of CIP-resistant genes in human subjects and swine *E. coli* isolates.

Humans (H)	Gene	Swine (S)	Gene
H.1.	*qnrS + qnrB + qnrA*	S.1.	*qnrS + qnrB*
H.2.	*qnrS + qnrB*	S.2.	*qnrS + qnrB*
H.3.	*qnrS + qnrB + qnrA*	S.3.	*qnrS + qnrB*
H.4.	*qnrS*	S.4.	*qnrS + qnrB*
H.5.	-	S.5.	*qnrS + qnrB*
H.6.	*qnrS*	S.6.	*qnrS + qnrB*
H.7.	*qnrS*	S.7.	*qnrS + qnrB*
H.8.	*qnrS + qnrB*	S.8.	*qnrS + qnrB*
H.9.	*qnrS*	S.9.	*qnrS + qnrB*
H.10.	*qnrS + qnrB +qnrA*	S.10.	*qnrS + qnrB*
H.11.	-	S.11.	*qnrS + qnrB*
H.12.	*qnrS + qnrB*	S.12.	*qnrS + qnrB*
H.13.	*qnrS + qnrB*	-	-
H.14.	*qnrS + qnrB*	-	-
H.15.	*qnrS + qnrB*	-	-

**Table 6:** Sequence primers used genes and fragments size.

Gene	Primer Used	Fragment Size
*qnrS*	F: ACGACATTCGTCAACTGGAA R: TTAATTGGCACCCTGTAGGC	417 bp
*qnrA*	F: ATTTCTCACGCCAGGATTTG R:GATCGGCAAAGGTTAGGTCA	516 bp
*qnrB*	F: GTTGGCGAAAAAATTGACAGAA R: ACTCCGAATTGGTCAGATCG	526 bp

## Abbreviations

Antimicrobial Resistance—AMR; Polymerase Chain Reaction—PCR; Ultraviolet radiation—UV; European Commission—EC; Population Correction Unit—PCU; ciprofloxacin—CIP; Antibiotic Susceptibility Testing—AST; base pairs—bp; human subjects—H; swine—S; Phosphate Buffered Saline—PBS; Optical Density—OD; Electrophoresis Buffer-Acetic Acid Buffer—EDTA-TAE; proteinase K—pk; quinolone resistant—qnr; Amoxicillin—AMX; Ampicillin—AMP; Cefalothin—CEF; Ciprofloxacin—CIP; Colistin—CST; Enrofloxacin—ENR; Florfenicol—FLO; Gentamicin—GEN; Lincomycin—LCM; Neomycin—NEO; Norfloxacin—NOR; Oxacillin—OXA; Potentiated sulfonamides—POS; Spectinomycin—SPCM; Tetracycline—TC; Human sample—H; Ampicillin-sulbactam—SAM; Cefepime—CFPM; Ceftazidime—CAZ; Ceftriaxone—CTX; Cefuroxime—CXM; Ciprofloxacin—CIP; Levofloxacin—LVX; Piperacillin—PIP; Piperacillin-tazobactam—TZP; Trimethoprim—TPM; Clinical Laboratory Standards Institute—CLSI.
